# Comparative Transcriptome Analysis of the Abdominal Ganglion Reveals Molecular Networks and Key Genes Underlying Thermal Tolerance in Red Swamp Crayfish (*Procambarus clarkii*)

**DOI:** 10.3390/ani16131988

**Published:** 2026-06-27

**Authors:** Beiqi Yang, Yue Ma, Qiujin Wang, Yi Liu, Liang Jia, Zhiyi Bai

**Affiliations:** 1 Key Laboratory of Freshwater Aquatic Genetic Resources, Ministry of Agriculture and Rural Affairs, Shanghai Ocean University, Shanghai 201306, China; yangbeiqi@126.com (B.Y.); 13348964792@163.com (Y.M.); wqj963260@163.com (Q.W.); 18861911625@163.com (Y.L.); 2 Shanghai Collaborative Innovation Center for Cultivating Elite Breeds and Green-Culture of Aquaculture Animals, Shanghai Ocean University, Shanghai 201306, China

**Keywords:** *Procambarus clarkii*, water temperature, comparative transcriptome, abdominal ganglion

## Abstract

Water temperature fluctuations directly affect the feeding, reproduction, and survival of aquatic animals. Understanding the molecular networks that regulate temperature responses is therefore critical for selective breeding of thermotolerant strains. In this study, we first assessed the thermotolerant performances of two *Procambarus clarkii* populations (selected and normal cultured populations) with contrasting thermal endurance capacities. Temperature stress challenges showed that the selected population exhibited great endurance capacities to both heat and cold stresses. Further comparative transcriptomic analysis showed that cold stress mainly upregulated energy metabolism genes, whereas heat stress affected oxidative stress and membrane-related genes in both populations. However, we found that immune-related pathway genes were significantly downregulated in the selected population in response to heat challenge. Furthermore, heat shock proteins (HSPs), E3 ubiquitination, and autophagy-related genes showed contrasting expression patterns between the thermotolerant and normal cultured populations, suggesting that transcriptional regulations of these signaling pathways may contribute to thermal tolerance. Collectively, these results provide a reference for breeding thermotolerant *P. clarkii* strains and supporting the sustainable development of the aquaculture industry.

## Simple Summary

Temperature significantly affects the physiology and survival of *Procambarus clarkii*. This study aims to comprehensively analyze the molecular basis of enhanced thermotolerance in *P. clarkii*. To achieve this, we integrated phenotypic analysis with comparative transcriptomic approaches. Our results showed that the selected population of *P. clarkii* also exhibits remarkable cold resistance. Comparative transcriptomic analysis of the abdominal ganglion identified a large number of differentially expressed genes (DEGs) that are primarily enriched in metabolic processes under low-temperature stress, whereas they are involved in protein folding processes under high-temperature stress. Comparative analysis further showed that under control conditions, metabolic pathways differ between the selected and normal cultured populations, while immune-related processes are significantly enriched in the selected population under temperature stress. Additionally, differential expression of temperature-responsive-related genes under high temperature stress suggests that they may serve as key targets for breeding heat-tolerant crayfish. These findings provide valuable insights into the temperature adaptation mechanisms of *P. clarkii*.

## 1. Introduction

Water temperature critically determines the survival, metabolism, reproduction, and development of aquatic animals [1]. To cope with thermal fluctuations, aquatic animals have evolved sophisticated molecular systems that convert environmental stimuli into physiological responses. This process begins when the peripheral nervous system detects temperature fluctuations via transient receptor potential (TRP) channels, triggering behavioral thermotaxis toward the optimal temperature zone [2,3]. However, when fluctuations exceed physiological limits, they cause protein misfolding and metabolic imbalance. To counteract this, aquatic animals remodel their biochemical homeostasis through a coordinated network of heat shock proteins (HSPs), E3 ubiquitination, and autophagy [4,5,6,7].

As molecular chaperones, HSPs are essential for protein folding and homeostasis under temperature stress [8]. The synergy between TRP channels and HSPs is bidirectional: TRP channel activation induces HSP expression, whereas certain HSPs, such as Hsc70, can retroactively modulate TRP channel sensitivity [9,10,11]. When thermal stress causes irreversible damage, the E3 ubiquitin–autophagy pathway is recruited to clear non-repairable proteins and organelles [12]. Together, these integrated pathways—from TRP-mediated sensing to HSP-mediated repair and autophagic degradation—form a robust defense system that enables aquatic animals to cope with thermal fluctuations.

The red swamp crayfish (*Procambarus clarkii*) is a cornerstone of freshwater aquaculture in China, consistently ranking first in the domestic crustacean production. However, the industry faces growing constraints from global warming and regional cultivation bottlenecks [13,14,15]. Unfavorable thermal stress induces deleterious behaviors in *P. clarkii*, such as excessive burrowing, which reduces feed intake, arrests growth, and increases mortality. While transcriptomic analyses have identified several stress-responsive pathways, including metabolism, protein processing, immunity, and redox regulation [16,17,18], these studies have primarily focused on peripheral organs like the hepatopancreas and gills. Consequently, the neural tissues responsible for thermal perception, specifically the abdominal ganglion, remain largely unexplored. In mammals, sensory neurons within the dorsal root ganglia are the primary thermal sensors [19]. Because temperature similarly regulates abdominal ganglion neural activity, investigating this tissue from a thermosensory perspective is critical for understanding the molecular mechanisms of thermal tolerance. Although our laboratory has successfully established a thermotolerant *P. clarkii* population through selective breeding [20], the molecular mechanisms underlying this enhanced resilience remain unclear.

To this end, this study used normal and selectively bred thermotolerant populations of *P. clarkii* as experimental subjects. By integrating phenotypic thermotolerance with transcriptome sequencing analysis, we compared the response differences between the two populations under high- and low-temperature stress. In addition, we screened candidate genes involved in temperature sensing, heat stress response, ubiquitination, autophagy, and other temperature-responsive processes. Together, this study revealed transcriptional regulatory differences between the two populations, providing a foundation for molecular breeding of thermotolerant *P. clarkii* strains.

## 2. Materials and Methods

### 2.1. Crayfish Acclimation and Thermotolerance Performance Assessment

The selected population (SP) of *Procambarus clarkii* used in this experiment was derived from a cross between female crayfish collected from Gehu Lake, Jiangsu Province, and male crayfish collected from Wuhu, Anhui Province. This hybrid population was then continuously bred and screened for high-temperature tolerance based on the Arrhenius breakpoint temperature (ABT) at a farm in Wuhu, Anhui Province, China, for successive generations until the F_4_ generation was established [20]. The normal population (NP) used in the experiment was a locally maintained, unselected farm population that had been reared at the same farm for at least three consecutive generations prior to the experiment. Prior to temperature stress exposure, all experimental crayfish were acclimated in experimental tanks for 48 h. During the acclimation period, the water temperature was maintained at 23 ± 0.5 °C, dissolved oxygen was >6.0 mg/L, pH was 7.2–7.6, and the photoperiod was set at 12L:12D.

The thermotolerance performance of *P. clarkii* was characterized using two protocols: chronic heating for critical thermal maximum (CTmax) assessment and acute heating for Arrhenius breakpoint temperature (ABT) assessment.

For CTmax assessment, healthy individuals (n = 30 per population) acclimated for 48 h were subjected to a chronic heating protocol. Starting at 26 °C, water temperature was raised at 1 °C per 2 h to 35 °C, followed by 0.5 °C per 4 h until loss of equilibrium. CTmax was calculated as the arithmetic mean of the temperatures at which each crayfish lost equilibrium. Loss of equilibrium was defined as the inability to maintain an upright posture, failure to right when turned over, and curled body posture. This condition was reversible upon return to room-temperature water.

Heart rate, a key indicator of metabolic status, exhibits a biphasic response to rising temperature: an initial increase followed by a gradual decline, with the inflection point defined as the ABT [21]. Originally established in bivalves and subsequently refined by our laboratory, ABT was assessed via acute heating [20,22]. Specifically, an infrared heart rate monitor probe was fixed to the posterior dorsal cephalothorax. After 1 h of acclimation, heartbeat signals were amplified and filtered (AMP03, Heartbeat Monitor, Newshift, Portugal), recorded via oscilloscope software (PowerLab 8/30, AD Instruments, Gilching, Germany), and heart rate (bpm) was calculated using LabChart Version 8.0. Each treatment group consisted of 30 individuals. ABT values were calculated using the following formula:

$$\ln\frac{k_{2}}{k_{1}}=-\frac{E_{a}}{R}\left(\frac{1}{T_{2}}-\frac{1}{T_{1}}\right),$$

where *k* is the number of cycles per minute (bpm), *T* is the absolute temperature (K), *E_a_* is the activation energy, and *R* is the universal gas constant, both of which are considered constants in this formula.

Water temperature was controlled using a temperature-controlled heater with an accuracy of 0.1 °C. Continuous aeration was maintained throughout the experiment to ensure uniform temperature distribution within the glass tanks.

### 2.2. Experimental Design and Sample Collection

A total of 500 healthy individuals of uniform size were randomly selected. Adult crayfish from the normal population had a body weight of 15.07 ± 2.77 g, and those from the selected population had a body weight of 15.26 ± 3.07 g. All animals showed no injury or disease and were not in the molting stage. Temperature stress was induced using a gradual temperature change scheme, with 23 °C as the starting point. The temperature was increased or decreased at a rate of 1 °C per 4 h until reaching 35 °C or 8 °C, followed by sustained stress for 12 h (for high temperature) or 24 h (for low temperature), respectively. Throughout the experiment, continuous aeration was provided, and no feed was supplied to avoid metabolic interference. A total of six treatment groups were established (Table 1), with three biological replicates per treatment group. Each replicate consisted of a pooled sample of abdominal ganglion from five individuals to minimize variation. After experimental treatment, crayfish were anesthetized on ice for 20 min. The abdominal ganglia were rapidly dissected and immersed in RNAlater solution. Samples were kept at room temperature overnight and then stored at −80 °C for further analysis.

### 2.3. RNA Extraction

Total RNA was extracted from each crayfish using TRIzol reagent (Invitrogen, Carlsbad, CA, USA) following the manufacturer’s protocol. RNA purity and integrity were assessed using a NanoDrop 2000 spectrophotometer (Thermo Fisher Scientific, Waltham, MA, USA) and an Agilent 5300 Bioanalyzer (Agilent Technologies, Santa Clara, CA, USA), respectively. For each biological replicate, equal amounts of RNA from the abdominal ganglia of five individuals were pooled. Samples with an RNA integrity number (RIN) ≥ 6.5 and A260/280 ratios of approximately 2.0 were used for subsequent library construction, which was conducted by Shanghai Majorbio Bio-Pharm Technology Co., Ltd. (Shanghai, China).

### 2.4. RNA Sequencing and Differential Expression Analysis

For RNA-seq, qualified RNA samples were used for mRNA enrichment with oligo(dT) beads and subsequent cDNA library construction. Sequencing was performed on the Illumina NovaSeq X Plus platform (Illumina, San Diego, CA, USA) with a paired-end 150 bp (PE150) read length, generating approximately 12 Gb of raw data per sample. Raw reads were processed using fastp (Version 0.23.4) to remove low-quality bases and adapters [23]. The clean reads were then aligned to the reference genome (GCF_040958095.1) using HISAT2 (version 2.2.1), and gene expression levels were quantified using RSEM (version 1.3.3) [24,25]. Differential expression analysis was conducted using DESeq2 (version 1.10.1), with genes meeting the criteria of an adjusted *p*-value (*P.*adj) < 0.05 and |log_2_ fold change (FC)| > 1 considered significantly differentially expressed [26].

### 2.5. Function Enrichment Analysis

To identify the biological functions of differentially expressed gene sets, including core response genes and population-specific genes, Gene Ontology (GO) enrichment analysis was performed using Goatools (version 0.6.5)with Fisher’s exact test [27]. To account for multiple testing and to control the false discovery rate, *p*-values were adjusted using the Benjamini–Hochberg (BH) method. GO terms with an adjusted *p*-value (*P.*adj) < 0.05 were considered significantly enriched. For KEGG pathway enrichment, the KOBAS online tool (http://bioinfo.org/kobas) (accessed on 6 March 2026) was employed, applying the same statistical approach as used in the GO enrichment analysis [28].

### 2.6. Quantitative PCR Validation of DEGs

To validate the accuracy of the RNA-Seq data, we selected 12 target genes for quantitative PCR (qPCR) validation. Specific primers were designed using Primer Premier 5.0 software (Table S1). Complementary DNA (cDNA) was synthesized from total RNA using the PrimeScript™ RT reagent Kit with gDNA Eraser (Takara Bio Inc., Kusatsu, Shiga, Japan). The 20 μL reaction system consisted of 6.8 μL RNase-free water, 1.6 μL cDNA, 0.8 μL each of forward and reverse primers, and 10 μL TB Green^®^ Premix Ex Taq™ II (Tli RNaseH Plus) (Takara Bio Inc., Kusatsu, Shiga, Japan). The reaction program was performed on a C1000™ Thermal Cycler (Bio-Rad, Hercules, CA, USA), as follows: initial denaturation at 95 °C for 30 s, followed by 40 cycles of denaturation at 95 °C for 5 s, annealing at 58 °C for 30 s, and extension for 30 s. *18S rRNA* was used as the internal reference gene, and relative expression levels were calculated using the 2^−ΔΔCt^ method. Each biological replicate included three technical replicates.

### 2.7. Statistical Analysis

Data were presented as mean ± standard deviation (mean ± SD). Differences among groups were evaluated using one-way analysis of variance (ANOVA) followed by Tukey’s HSD test, with the significance level set at *p* < 0.05. Related graphs and charts were generated using GraphPad Prism 9.5.

## 3. Results

### 3.1. Selected Population of Procambarus clarkii Exhibits Bidirectional Thermal Adaptability

**Figure 1:**
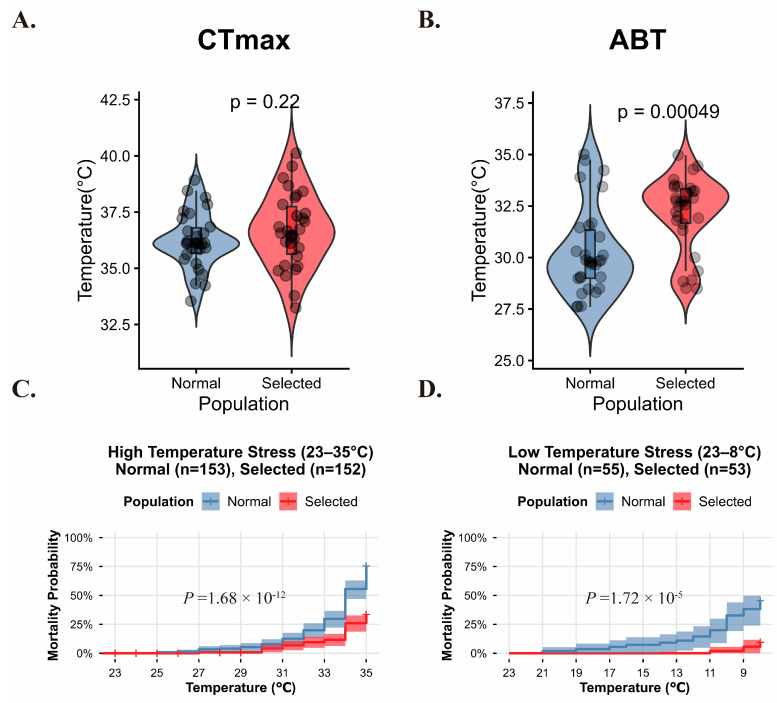
Evaluation of temperature resistance in selected and normal populations of *P. clarkii*. (**A**) CTmax and (**B**) ABT of selected and normal populations of *P. clarkii* (n = 30). Mortality curves of selected and normal populations under (**C**) high and (**D**) low temperature stress. *p*-values were calculated using the log-rank (Mantel–Cox) test to compare mortality curves between the selected and normal populations.

We first employed a combination of chronic and acute heating experiments to evaluate the thermotolerance performance of two populations of *Procambarus clarkii*, which were a selected population and a normal population. The results showed that the critical thermal maximum (CTmax) of the selected population was higher than that of the normal population, although the difference failed to reach statistical significance (Figure 1A). In contrast, the Arrhenius breakpoint temperature (ABT) of the selected population was significantly higher than that of the normal population, indicating enhanced high-temperature adaptability (Figure 1B).

To further validate the differences in thermal adaptation between the two populations, a temperature stress experiment was conducted with three treatment groups: low-temperature stress (8 °C), control (23 °C), and high-temperature stress (35 °C). Under high-temperature stress, the mortality rate of the selected population was significantly lower than that of the normal population, indicating higher thermotolerance in the selected population based on ABT and mortality (Figure 1C). Under low-temperature stress, the selected population also exhibited a significantly lower mortality rate compared with the normal population (Figure 1D).

### 3.2. Temperature Stress Induces Population-Specific Transcriptional Divergence in P. clarkii

To assess RNA-seq data quality and characterize the transcriptional response to temperature stress, we sequenced 18 cDNA libraries from temperature-stressed *P. clarkii*. After quality control, raw reads per sample ranged from 60.8 to 77.7 million, and clean reads ranged from 60.8 to 77.1 million, with a mean error rate of 0.0118–0.0120%. The Q30 scores, representing the percentage of bases with a Phred quality score ≥ 30, ranged from 96.00% to 96.40%, indicating high base-calling accuracy. GC content was consistent across samples (40.45–42.95%), indicating no significant nucleotide composition bias. Furthermore, uniquely mapped reads accounted for 74.7–81.9% of clean reads, demonstrating sufficient mapping specificity for differential expression analysis (Table 2). Collectively, these results confirm that the data are of sufficient quality and depth for subsequent analyses.

**Figure 2:**
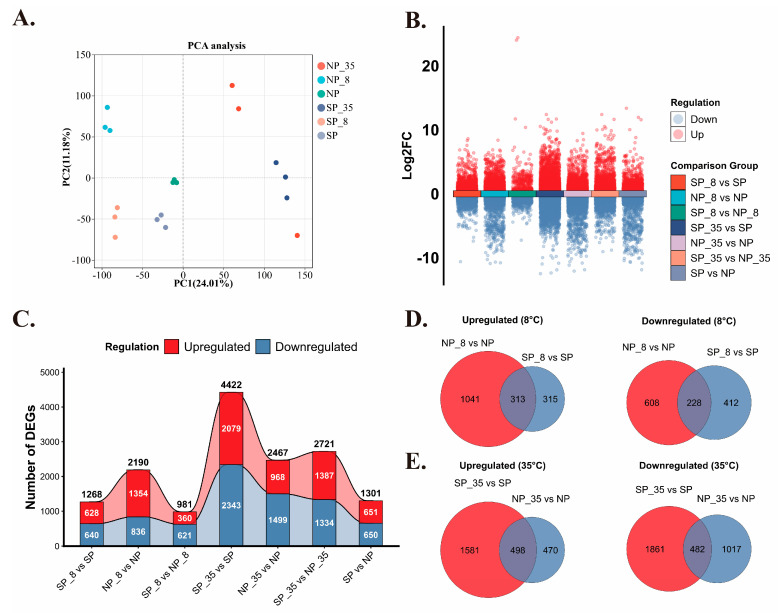
Identification of DEGs. (**A**) PCA plot of transcriptome profiles. (**B**) Volcano plot of DEGs; (**C**) statistics of DEGs. Venn diagrams show (**D**) conserved upregulated and downregulated DEGs in both populations after low-temperature stress, and (**E**) conserved upregulated and downregulated DEGs in both populations after high-temperature stress. Sample groups: NP, normal population at 23 °C; NP_8, normal population at 8 °C; NP_35, normal population at 35 °C; SP, selected population at 23 °C; SP_8, selected population at 8 °C; SP_35, selected population at 35 °C. Same for subsequent figures.

Principal component analysis (PCA) showed good within-group reproducibility for all samples except NP_35_3, which was therefore excluded from subsequent analyses (Figure 2A). Under control conditions, the two populations already showed some separation. Following low- and high-temperature stress, samples from each population became more distinctly separated. These results indicate that the transcriptional responses to temperature stress differ markedly between the two populations.

### 3.3. Temperature Stress Induces Population-Specific DEG Patterns in P. clarkii

To investigate the conserved mechanisms of temperature stress response and the molecular basis of thermotolerance differences between the two populations, we established seven comparison groups: TP_8 vs. TP, NP_8 vs. NP, TP_8 vs. NP_8, TP_35 vs. TP, NP_35 vs. NP, TP_35 vs. NP_35, and TP vs. NP. Differentially expressed genes (DEGs) were identified by comparing transcript levels under temperature stress with those in the corresponding control groups, using a threshold of fold change ≥ 2.0 or fold change ≤ 0.5 and *P*.adj < 0.05 (Figure 2B).

In the normal population, low-temperature exposure induced 2190 DEGs (1354 up, 836 down), whereas high-temperature exposure induced 2467 DEGs (968 up, 1499 down). In the selected population, low-temperature treatment induced 1268 DEGs (628 up, 640 down), whereas high-temperature treatment induced 4422 DEGs (2079 up, 2343 down) (Figure 2C). Notably, high-temperature stress triggered a stronger transcriptional response in the selected population. However, given the substantial baseline differences between the two populations under control conditions (1301 DEGs at 23 °C), the higher DEG count in the selected population may partly reflect pre-existing divergence rather than a stronger stress response.

Under control conditions, we detected 1301 DEGs between the two populations, with comparable numbers of up- and downregulated genes (651 and 650, respectively). Following low-temperature exposure, the number of interpopulation DEGs decreased to 981 (360 up, 621 down). In contrast, under high-temperature stress, the interpopulation differences expanded substantially to 2721 DEGs (1387 upregulated, 1334 downregulated) (Figure 2C).

Venn diagram analysis showed that under low temperature, 313 DEGs were commonly upregulated and 228 DEGs were commonly downregulated in both populations (Figure 2D). Under high temperature, 498 DEGs were commonly upregulated and 482 commonly downregulated across the two populations (Figure 2E). Common DEGs accounted for 14.35–19.5% of the total, indicating substantial differences in transcriptional responses between the two populations. These common DEGs, which showed consistent expression patterns in both populations, represent conserved transcriptional regulatory mechanisms underlying temperature stress response in *P. clarkii.* Together, these results suggest that although both populations share a core set of conserved temperature-responsive genes, most transcriptional changes are population-specific, providing a molecular basis for their differential thermotolerance.

### 3.4. GO and KEGG Function Enrichment

To investigate the conserved pathways underlying the response to temperature stress in *P. clarkii* and to explore population-specific mechanisms, we performed GO and KEGG enrichment analyses on the common DEGs identified in both populations, as well as on the DEGs in both the thermotolerant and normal populations.

#### 3.4.1. Low Temperature Affects the Energy Metabolism-Related Genes in *P. clarkii*

**Figure 3:**
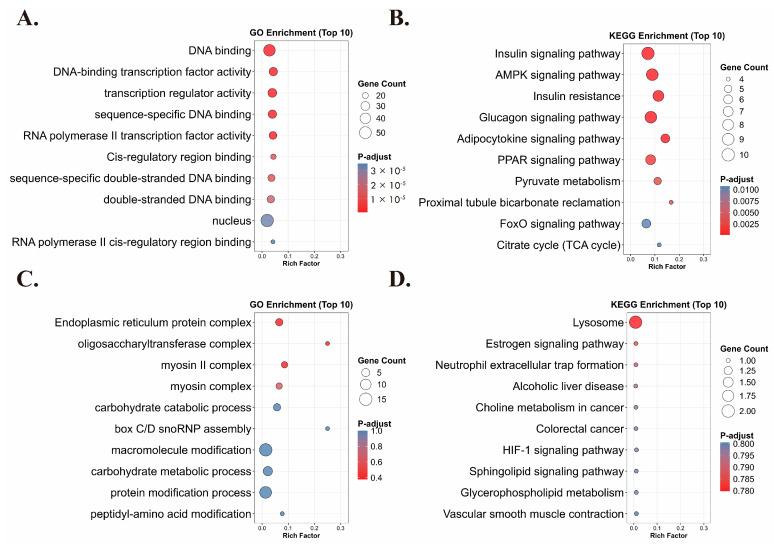
GO and KEGG enrichment analyses of conserved low-temperature-responsive DEGs. (**A**) GO terms and (**B**) KEGG pathways enriched among the commonly upregulated DEGs in the TP_8 vs. TP and NP_8 vs. NP comparisons. (**C**) GO terms and (**D**) KEGG pathways enriched among the commonly downregulated DEGs in the TP_8 vs. TP and NP_8 vs. NP comparisons.

GO enrichment analysis revealed that the commonly upregulated DEGs in both populations under low-temperature stress were significantly enriched in transcriptional regulation-related GO terms, including DNA binding (GO:0003677, *P.*adj = 5.47 × 10^−9^), DNA-binding transcription factor activity (GO:0003700, *P.*adj = 2.30 × 10^−8^), transcription regulator activity (GO:0140110, *P.*adj = 2.61 × 10^−8^), sequence-specific DNA binding (GO:0043565, *P.*adj = 7.87 × 10^−8^), and RNA polymerase II transcription factor activity (GO:0000981, *P.*adj = 2.30 × 10^−7^) (Figure 3A). KEGG enrichment analysis showed that the commonly upregulated DEGs were primarily enriched in pathways involved in energy metabolism and the regulation of glucose and lipid metabolism, including the insulin signaling pathway (map04910, *P.*adj = 1.53 × 10^−4^), AMPK signaling pathway (map04152, *P.*adj = 1.55 × 10^−4^), PPAR signaling pathway (map03320, *P.*adj = 1.29 × 10^−3^), and FoxO signaling pathway (map04068, *P.*adj = 1.06 × 10^−2^) (Figure 3B). For commonly downregulated DEGs, neither GO nor KEGG enrichment analysis yielded statistically significant results (Figure 3C,D). These results suggest that low-temperature stress induces coordinated upregulation of transcriptional regulators and metabolic signaling pathways involved in energy homeostasis in *P. clarkii*.

#### 3.4.2. High Temperature Affects Protein Folding and Oxidative Stress-Related Genes in *P. clarkii*

**Figure 4:**
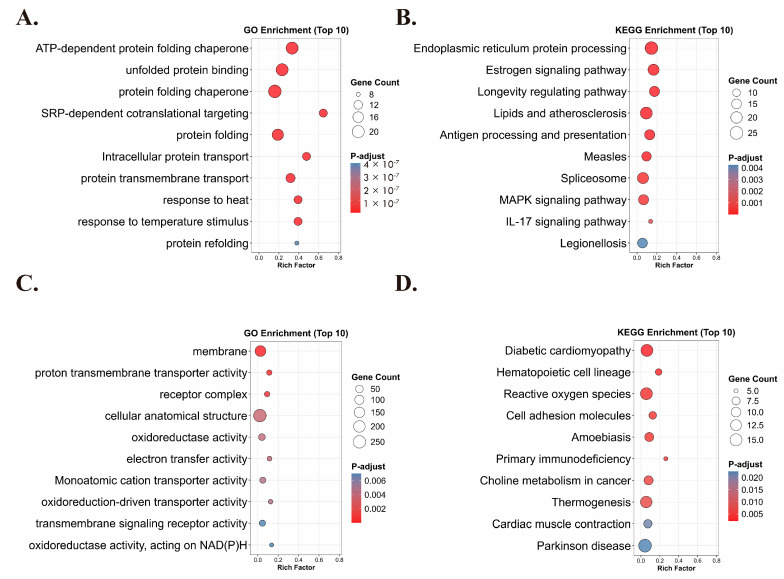
GO and KEGG enrichment analyses of conserved high temperature-responsive DEGs. (**A**) GO terms and (**B**) KEGG pathways enriched among the commonly upregulated DEGs in the TP_35 vs. TP and NP_35 vs. NP comparisons. (**C**) GO terms and (**D**) KEGG pathways enriched among the commonly downregulated DEGs in the TP_35 vs. TP and NP_35 vs. NP comparisons.

GO enrichment analysis of the commonly upregulated DEGs under high-temperature stress revealed significant enrichment in terms associated with protein folding, such as ATP-dependent protein folding chaperone (GO:0140662, *P.*adj = 3.47 × 10^−18^), unfolded protein binding (GO:0051082, *P.*adj = 5.23 × 10^−15^), protein folding chaperone (GO:0044183, *P.*adj = 3.74 × 10^−13^), SRP-dependent cotranslational targeting (GO:0006616, *P.*adj = 7.11 × 10^−13^), and protein folding (GO:0006457, *P.*adj = 2.13 × 10^−11^) (Figure 4A). Consistent with the GO results, KEGG enrichment analysis identified significant enrichment of protein processing in the endoplasmic reticulum pathway (map04141, *P.*adj = 3.18 × 10^−13^), estrogen signaling pathway (map04915, *P*.adj = 3.67 × 10^−11^), longevity regulating pathway (map04213, *P*.adj = 8.21 × 10^−10^), lipids and atherosclerosis (map05417, *P*.adj = 3.38 × 10^−8^), and the antigen processing and presentation pathway (map04612, *P.*adj = 6.69 × 10^−8^) (Figure 4B).

For commonly downregulated DEGs under high-temperature stress, GO analysis showed significant enrichment in the membrane (GO:0016020, *P.*adj = 5.13 × 10^−7^), proton transmembrane transporter activity (GO:0015078, *P.*adj = 2.33 × 10^−4^), receptor complex (GO:0043235, *P.*adj = 5.35 × 10^−4^), cellular anatomical structure (GO:0110165, *P.*adj = 3.72 × 10^−3^), and oxidoreductase activity (GO:0016491, *P.*adj = 4.03 × 10^−3^) (Figure 4C). In agreement with the GO findings, KEGG enrichment analysis showed that the DEGs were significantly enriched in pathways associated with membrane structure and oxidative stress, including diabetic cardiomyopathy (map05415, *P.*adj = 2.17 × 10^−3^), hematopoietic cell lineage (map04640, *P.*adj = 2.63 × 10^−3^), reactive oxygen species (map05208, *P.*adj = 3.80 × 10^−3^), cell adhesion molecules (map04514, *P.*adj = 4.15 × 10^−3^), and amoebiasis (map05146, *P.*adj = 4.15 × 10^−3^) (Figure 4D). These results indicate that under high-temperature stress, the commonly upregulated DEGs in both populations were significantly enriched in terms associated with protein homeostasis, including protein folding and endoplasmic reticulum processing pathways, whereas the commonly downregulated DEGs were significantly enriched in membrane-associated and oxidoreductase activities.

#### 3.4.3. Baseline Transcriptional Differences Between Selected and Normal Populations

**Figure 5:**
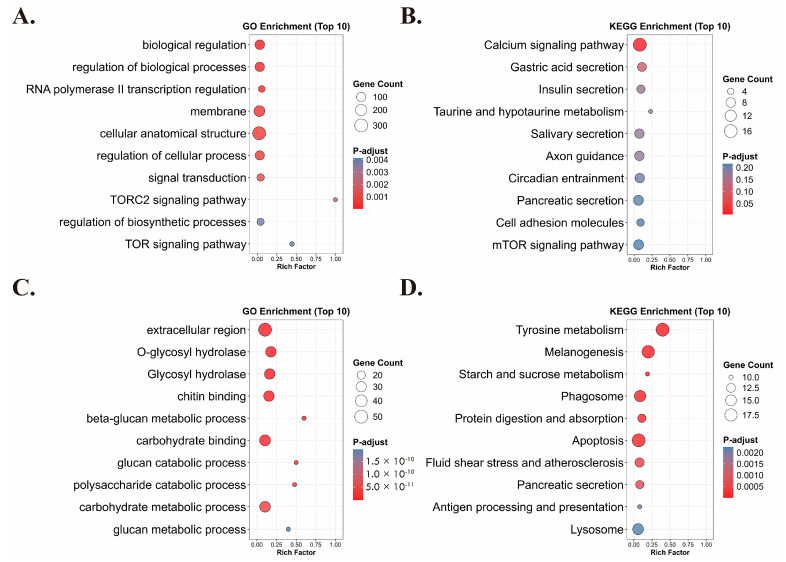
GO and KEGG enrichment analyses of DEGs between the selected and normal populations under control conditions. (**A**) GO terms and (**B**) KEGG pathways enriched among upregulated DEGs in the selected population compared to the normal population (SP vs. NP). (**C**) GO terms and (**D**) KEGG pathways enriched among downregulated DEGs in the selected population compared to the normal population (SP vs. NP).

To elucidate the molecular basis of thermotolerance in *P. clarkii*, we analyzed baseline gene expression differences between the thermotolerant and normal populations under control conditions, as well as their differential responses to temperature stress. Under control conditions, KEGG and GO enrichment analyses showed that upregulated genes were significantly enriched in biological regulation (GO:0065007, *P.*adj = 2.45 × 10^−5^), regulation of biological processes (GO:0050789, *P.*adj = 1.84 × 10^−4^), RNA polymerase II transcription regulation (GO:0006357, *P.*adj = 2.18 × 10^−4^), in the membrane (GO:0016020, *P.*adj = 4.29 × 10^−4^), cellular anatomical structure (GO:0110165, *P.*adj = 5.70 × 10^−4^), and the calcium signaling pathway (map04020, *P.*adj = 4.27 × 10^−3^) (Figure 5A,B). In contrast, downregulated genes were significantly enriched in the extracellular region (GO:0005576, *P.*adj = 1.99 × 10^−17^), O-glycosyl hydrolase (GO:0004553, *P.*adj = 4.66 × 10^−17^), glycosyl hydrolase (GO:0016798, *P.*adj = 3.93 × 10^−16^), chitin binding (GO:0008061, *P.*adj = 1.62 × 10^−15^), the beta-glucan metabolic process (GO:0051273, *P.*adj = 6.38 × 10^−13^), tyrosine metabolism (map00350, *P.*adj = 1.01 × 10^−18^), melanogenesis (map04916, *P*.adj = 5.74 × 10^−12^), starch and sucrose metabolism (map00500, *P.*adj = 2.77 × 10^−6^), phagosome (map04145, *P*.adj = 2.33 × 10^−5^), and protein digestion and absorption (map04974, *P*.adj = 3.49 × 10^−5^) (Figure 5C,D). These results indicate that under control conditions, the selected population exhibits a significant upregulation of genes involved in transcriptional regulation and calcium signaling, alongside downregulation of genes associated with metabolic and extracellular matrix processes, suggesting a distinct baseline regulatory landscape that may contribute to its enhanced thermal adaptability.

#### 3.4.4. Low Temperature Downregulates Immune-Related Genes in the Selected Population

**Figure 6:**
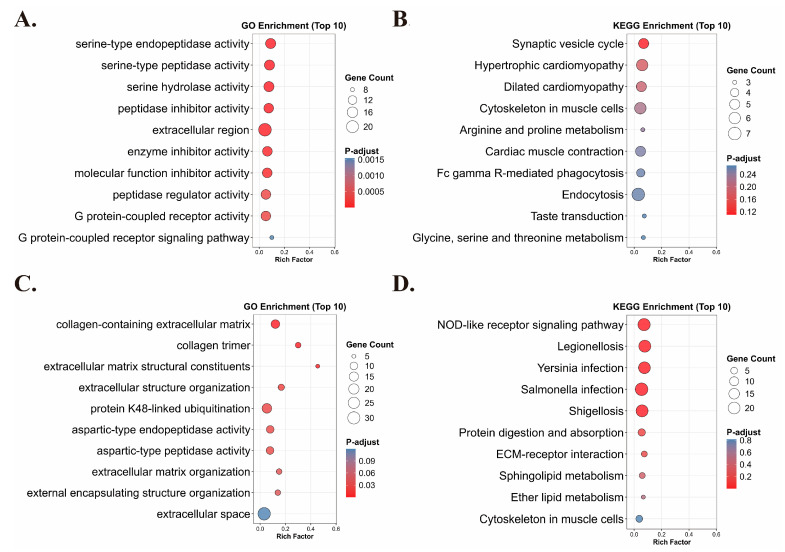
GO and KEGG enrichment analyses of DEGs between the selected and normal populations under low-temperature conditions. (**A**) GO terms and (**B**) KEGG pathways enriched among upregulated DEGs in the selected population compared to the normal population (SP_8 vs. NP_8). (**C**) GO terms and (**D**) KEGG pathways enriched among downregulated DEGs in the selected population compared to the normal population (SP_8 vs. NP_8).

Following low-temperature stress, KEGG and GO enrichment analyses revealed that upregulated genes were significantly enriched in GO terms related to serine proteases, including serine-type endopeptidase activity (GO:0004252, *P.*adj = 8.33 × 10^−7^), serine-type peptidase activity (GO:0008236, *P.*adj = 1.91 × 10^−6^), serine hydrolase activity (GO:0017171, *P.*adj = 2.84 × 10^−6^), peptidase inhibitor activity (GO:0030414, *P.*adj = 9.57 × 10^−6^), and in the extracellular region (GO:0005576, *P.*adj = 1.49 × 10^−5^) (Figure 6A). However, no KEGG pathways were significantly enriched among these DEGs (Figure 6B). Downregulated genes were significantly enriched in the collagen-containing extracellular matrix (GO:0062023, *P.*adj = 2.13 × 10^−5^), collagen trimer (GO:0005581, *P.*adj = 2.83 × 10^−4^), extracellular matrix structural constituents (GO:0030020, *P.*adj = 3.35 × 10^−4^), extracellular structure organization (GO:0043062, *P.*adj = 2.05 × 10^−2^), protein K48-linked ubiquitination (GO:0070936, *P*.adj = 2.53× 10^−2^), the NOD-like receptor signaling pathway (map04621, *P.*adj = 6.92 × 10^−8^), legionellosis (map05134, *P.*adj = 9.33 × 10^−8^), yersinia infection (map05135, *P.*adj = 1.48 × 10^−8^), salmonella infection (map05132, *P.*adj = 1.69 × 10^−6^), and shigellosis (map05131, *P.*adj = 3.56 × 10^−6^) (Figure 6C,D). These results suggest that under low-temperature stress, the downregulated DEGs in the selected population were significantly enriched in the NOD-like receptor and bacterial infection pathways.

#### 3.4.5. High Temperature Downregulates Immune and Neural Signaling Pathway-Related Genes in the Selected Population

**Figure 7:**
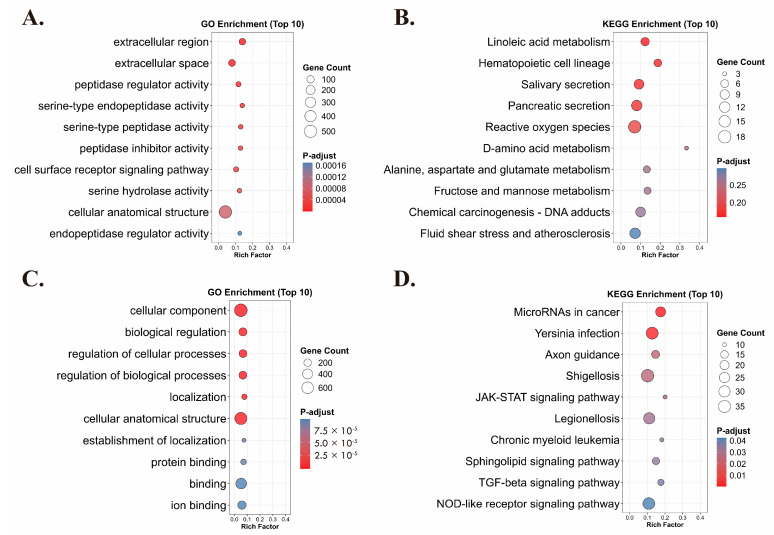
GO and KEGG enrichment analyses of DEGs between the selected and normal populations under high-temperature conditions. (**A**) GO terms and (**B**) KEGG pathways enriched among upregulated DEGs in the selected population compared to the normal population (SP_35 vs. NP_35). (**C**) GO terms and (**D**) KEGG pathways enriched among downregulated DEGs in the selected population compared to the normal population (SP_35 vs. NP_35).

Following high temperature stress, GO analysis showed significant enrichment in the extracellular region (GO:0005576, *P.*adj = 1.83 × 10^−19^), extracellular space (GO:0005615, *P.*adj = 2.98 × 10^−8^), peptidase regulator activity (GO:0061134, *P*.adj = 5.46 × 10^−6^), serine-type endopeptidase activity (GO:0004252, *P*.adj = 1.44 × 10^−5^), and serine-type peptidase activity (GO:0008236, *P*.adj = 2.12 × 10^−5^) (Figure 7A). For upregulated DEGs, KEGG enrichment showed linoleic acid metabolism (map00591, *P*.adj = 0.159), hematopoietic cell lineage (map04640, *P*.adj = 0.167), salivary secretion (map04970, *P*.adj = 0.168), pancreatic secretion (map04972, *P*.adj = 0.176), and chemical carcinogenesis—reactive oxygen species (map05208, *P*.adj = 0.193) were enriched, but showed no statistical significance (*P*.adj > 0.05) (Figure 7B). GO enrichment analysis of downregulated DEGs revealed significant enrichment in cellular components (GO:0005575, *P.*adj = 1.78 × 10^−9^), biological regulation (GO:0065007, *P.*adj = 1.13 × 10^−8^), regulation of cellular processes (GO:0050794, *P.*adj = 3.58 × 10^−8^), regulation of biological processes (GO:0050789, *P.*adj = 5.44 × 10^−8^), and localization (GO:0051179, *P.*adj = 1.24 × 10^−6^) (Figure 7C). KEGG enrichment analysis showed significant enrichment of the axon guidance (map04360, *P.*adj = 2.38 × 10^−2^), JAK-STAT signaling pathway (map04630, *P.*adj = 2.65 × 10^−2^), sphingolipid signaling pathway (map04071, *P.*adj = 3.50 × 10^−2^), TGF-beta signaling pathway (map04350, *P.*adj = 3.80 × 10^−2^), and NOD-like receptor signaling pathway (map04621, *P.*adj = 4.24 × 10^−2^) (Figure 7D). These results indicate that under high-temperature stress, the downregulated DEGs in the selected population were significantly enriched in immune-related pathways as well as the neural signaling pathway. In contrast, upregulated DEGs were associated with the extracellular region and serine protease activities, although their functional significance remains to be determined.

### 3.5. Differential Expression Patterns of Temperature-Responsive Genes Between the Two Populations in P. clarkii

Through differential expression analysis, we identified twenty-seven *HSP* genes, seven *TRP* genes, eleven autophagy-associated genes, twelve E3 ubiquitin ligase genes, and twenty-four transcription factor genes as candidates involved in temperature stress responses and thermotolerance acquisition. Expression patterns of these genes varied markedly with temperature stress and population genetic background.

#### 3.5.1. *TRP* Genes

**Figure 8:**
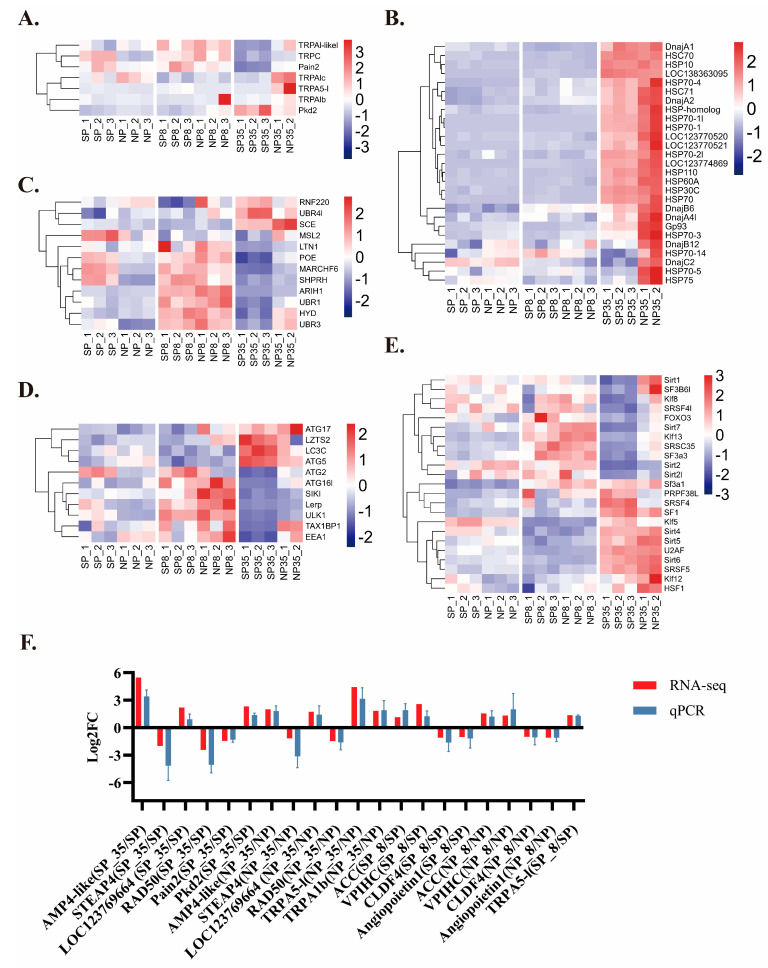
Expression patterns of key genes involved in the response to temperature stress revealed by transcriptomic analysis. (**A**) *TRP* genes; (**B**) *HSP* genes; (**C**) E3 ubiquitin ligase genes; (**D**) autophagy-associated genes; (**E**) temperature-associated transcription factors. All expression values were normalized by z-score across rows for each panel. In the heatmap, red indicates higher relative expression, blue indicates lower relative expression, and white represents the mean expression level. Each column represents a biological replicate. (**F**) Validation of RNA-seq data by qPCR analysis of 12 genes. Data are shown as mean ± SD of three biological replicates (n = 3).

Transient receptor potential (TRP) channels act as molecular sensors for temperature perception. We identified seven differentially expressed TRP genes in the *P. clarkii* transcriptome, spanning the TRPA, TRPC, and TRPP subfamilies. Under low-temperature stress, *TRP* gene expression patterns were similar between the two populations, whereas under control and high-temperature conditions, they diverged. Under control conditions, the selected population showed higher expressions of *TRPC* and *Pain2* but a lower expression of *TRPA1c* than the normal population. After high-temperature treatment, the selected population showed lower expressions of *TRPC*, *Pain2*, *TRPA1-like1*, *TRPA1c*, and *TRPA5-1* than the normal population, whereas *Pkd2* showed the opposite trend. No significant change was observed for *TRPA1b* (Figure 8A).

#### 3.5.2. *HSP* Genes

HSPs are conserved molecular chaperones that protect cells from various stresses, including temperature stress. Heatmap analysis showed that expression patterns of the two populations were clearly separated under high-temperature and control conditions, but similar under low-temperature stress. Specifically, all *HSP* genes were significantly upregulated after high-temperature exposure. Under low-temperature stress, *DnajA2* and LOC123774869 were significantly upregulated, whereas *Gp93* was significantly downregulated (Figure 8B). Comparative analysis showed that under high-temperature stress, *HSP* expression was significantly higher in the normal population than in the selected population. Under control conditions, *DnajB12* expression was significantly lower in the selected population than in the normal population.

#### 3.5.3. E3 Ubiquitin Ligase Genes

E3 ubiquitin ligases play critical roles in adaptation to temperature stress by mediating the degradation of temperature-sensitive proteins. Under low-temperature stress, the two populations showed similar expression patterns, with most genes significantly upregulated except *RNF220*, *MSL2*, *UBR4l*, and *SCE*. Under high-temperature and control conditions, however, the two populations showed clearly separated expression patterns (Figure 8C).

#### 3.5.4. Autophagy-Associated Genes

Under high-temperature stress, most autophagy-associated genes showed lower expression in the selected population than in the normal population, except for *LC3C*, *LZTS2*, and *ATG5*. Under low-temperature stress, most genes were significantly upregulated in both populations. Under control conditions, *ATG2*, *Lerp*, and *ULK1* were significantly higher in the selected population than in the normal population (Figure 8D).

#### 3.5.5. Key Transcription Factor Genes

Transcription factors act as molecular switches in gene expression and occupy central positions in regulatory networks that mediate responses to environmental temperature changes. Three classes of regulatory factors—Krüppel-like factors (Klfs), sirtuins (Sirts), and splicing factors (SFs)—coordinately mediate crustacean adaptive responses to high and low temperatures by regulating cell cycle progression, metabolic homeostasis, and alternative RNA splicing. Comparative analysis showed that transcription factor expression patterns were similar between the two populations under low-temperature stress but diverged under control and high-temperature conditions. Under control conditions, the selected population showed a significantly higher expression of *HSF1* and *FOXO3* than the normal population (Figure 8E). Under high-temperature stress, most transcription factors showed a significantly lower expression in the selected population, whereas *Sirt6*, *SRSF5*, *U2AF*, *SF1*, and *SF3a1* were significantly upregulated, and *Sirt2* and *Sirt2l* significantly downregulated (Figure 8E).

### 3.6. qPCR Validation of RNA-Seq Data

To validate the RNA-seq data, we selected 12 target genes for qPCR validation. Expression levels were normalized to *18S rRNA* and presented as fold change relative to this reference gene. Our results showed that the expression levels of all genes were consistent with RNA-seq trends, validating the transcriptomic data (Figure 8F).

## 4. Discussion

The ganglion is highly sensitive to temperature changes and exhibits rapid, dynamic responses to thermal stimuli [29,30,31]. Previous studies have shown that low temperature significantly affects energy, lipid, and amino acid metabolism in the gills, hemolymph, and muscles of *P. clarkii*, whereas high temperature influences energy metabolism, antioxidant defense, and immune processes in the hemolymph and hepatopancreas [16,17,18,32,33]. However, the response patterns of neural tissues remain poorly understood. We previously established a thermotolerant *P. clarkii* population through selective breeding [20]. Here, we first evaluated the thermotolerance of the two populations by measuring CTmax and ABT. The selected population showed significantly higher ABT than the normal population. To directly assess thermal tolerance, we conducted temperature stress experiments. These experiments revealed that the selected population had a significantly lower mortality than the normal population under both high and low temperatures. However, because our experimental design did not include a control group maintained constantly at 23 °C, we cannot attribute this mortality difference solely to temperature stress. Importantly, the selected population also showed enhanced cold survival, suggesting that selective breeding for heat tolerance may confer bidirectional thermal tolerance. Similar observations have been reported in other aquatic organisms, where selection for one thermal extreme enhances fitness at the opposite extreme [34].

To investigate conserved mechanisms of temperature stress responses and identify molecular targets for improving heat tolerance, we performed comparative transcriptome analysis on the two populations. We compared abdominal ganglion transcriptomes across temperature treatments and populations. We sequenced 18 samples from two populations under three temperature conditions. Raw data have been deposited in the NCBI Sequence Read Archive (accession number: PRJNA1442656).

To explore conserved transcriptional responses to temperature stress, we first identified commonly regulated DEGs in both populations. Under low-temperature stress, co-upregulated DEGs were significantly enriched in transcriptional regulation and energy metabolism pathways, including AMPK, PPAR, and FoxO signaling. AMPK functions as a cellular energy sensor activated upon ATP depletion. Once activated, AMPK restores energy homeostasis by promoting catabolism and suppressing anabolism [35,36]. The PPAR family includes nuclear receptor transcription factors that primarily regulate lipid-metabolism-related gene expression; their activation enhances fatty acid oxidation, lipid transport, and lipolysis to support energy production [37,38]. FoxO transcription factors regulate diverse biological processes, including oxidative stress responses, gluconeogenesis, and autophagy [39,40]. Under stress conditions, FoxO activation promotes expression of antioxidant enzymes, gluconeogenic enzymes, and autophagy-related genes, thereby enhancing cellular stress tolerance. Coordinated activation of these pathways likely mediates the metabolic adaptation of *P. clarkii* to low-temperature stress. Although AMPK and FoxO can also integrate cold stress signals, they were not significantly enriched in our heat stress data. These results showed that the enhanced cold tolerance of the selected line is not explained by shared enrichment of these metabolic pathways under heat stress.

Under high-temperature stress, DEGs co-downregulated in the two populations were primarily associated with proton transmembrane transporter activity and oxidoreductase activity. These results indicate that the downregulated genes are related to membrane-associated functions; however, the underlying mechanisms require further investigation. Such impairment may affect cell signaling, energy metabolism, and intercellular communication. Similar observations have been reported in other aquatic species, where heat stress downregulated genes involved in membrane transport and antioxidant defense [39,40].

To elucidate pathways underlying enhanced thermotolerance, we compared transcriptomic differences between the two populations under different temperatures. Under control conditions, downregulated DEGs in the selected population were significantly enriched in carbohydrate metabolism pathways, whereas upregulated DEGs were predominantly associated with membrane-associated biological regulation and the calcium signaling pathway. TRP channels, which serve as membrane-bound sensors of temperature and mediators of calcium influx, may represent key candidate factors of thermotolerance differences between the two populations.

Following high-temperature stress, downregulated DEGs in the selected population were significantly enriched in the JAK-STAT, TGF-β, and NOD-like receptor (NLR) signaling pathways. Downregulation of multiple immune-associated signaling pathways raises the possibility that the selected population adopts an immunosuppressive strategy under high-temperature stress. Such immunosuppression may represent an energy conservation mechanism that redirects resources from immune activation to essential survival processes. Furthermore, excessive inflammatory responses can cause tissue damage; thus, suppressing these pathways may also protect against heat-induced immunopathology [41]. Interestingly, under low-temperature stress, downregulated DEGs in the selected population were also significantly enriched in the NLR signaling pathway. As a key component of innate immunity, the NLR pathway senses cellular stress and damage and typically triggers inflammatory responses [42]. Downregulation of this pathway in the selected population under both high and low temperatures suggests that suppressing excessive inflammation may be a conserved bidirectional thermal adaptation strategy in crustaceans.

To identify molecular targets associated with thermal tolerance in *P. clarkii*, we examined the expression patterns of several gene families, including *HSP*s, *TRP*s, autophagy-related genes, and transcription factors. TRP channels are nonselective cation channels localized to the cell membrane. They convert external temperature stimuli into electrical signals, initiating downstream signaling cascades that mediate adaptive responses to temperature fluctuations [43]. In insects, homologs of *TRPA1*, *TRPA5*, *Pain2*, and *Pkd2* function as thermosensitive channels involved in temperature perception [44,45,46,47]. Here, seven *TRP* genes were differentially expressed between the two populations under high temperature stress. *Patinopecten yessoensis TRPA1-like* mediates calcium influx under high temperature and plays important roles in unfolded protein response (UPR) and apoptosis [48].

HSPs are major molecular chaperones that facilitate the clearance or degradation of misfolded proteins. Upon high-temperature stress, their expression rises sharply, exerting a protective effect. Here, *HSP* expression was markedly induced under heat stress. Notably, *HSP* expression was significantly higher in the normal population than in the selected population. Specifically, several HSP genes (including *HSP70-5*, *DnajC2*, *DnajB12*, *HSP75*, and *HSP70-14*) and their master transcriptional activator *HSF1* were downregulated or unchanged in the selected population, suggesting that strong induction of the classical heat shock response is not required for enhanced thermal tolerance [49].

E3 ubiquitin ligases regulate autophagy via protein ubiquitination and influence thermal tolerance by modulating key regulatory proteins [50,51]. Here, E3 ubiquitin ligase transcript levels were significantly lower in the selected population under heat stress, whereas both populations showed similar upregulation under cold stress. Autophagy-related genes showed similar expression patterns. ATG5 mediates LC3 lipidation via the ATG12-ATG5-ATG16L1 complex, regulating autophagosome formation and autophagic flux [52,53]. Specific upregulation of *ULK1*, *ATG5*, and LC3C in the selected population suggests a potential role for autophagy in thermotolerance, consistent with reports that *ATG5* enhances heat tolerance in tomato *Solanum lycopersicum* and *ULK1* in *Tetranychus urticae* [54,55]. Together, these findings suggest that E3 ubiquitin-mediated processes and autophagy may be involved in population differences in thermotolerance, although the precise mechanisms remain unclear. Most E3 ubiquitin ligase genes showed lower transcript levels in the selected population under heat stress. This seemingly paradoxical observation has two non-exclusive explanations. First, the selected population may have a lower baseline demand for stress-induced protein turnover, possibly due to more efficient protein folding or reduced protein damage under heat stress. Second, E3 ligase activities are tightly regulated post-translationally (e.g., by autoubiquitination or phosphorylation), which transcriptomic analysis alone cannot capture. Thus, lower transcript levels do not necessarily indicate lower functional activity.

Transcription factors activate or repress gene expression by recruiting regulatory elements to promoter regions. Expression profiling showed that temperature markedly reshaped the transcription factor landscape in *P. clarkii*: under high temperature, transcription factor expression was significantly lower in the selected population than in the normal population, whereas under low-temperature and control conditions, the two populations showed similar expression patterns.

Sirtuins (Sirts) are highly conserved NAD^+^-dependent deacetylases that regulate diverse substrate proteins by removing acetyl groups from lysine residues [56]. Previous studies have shown that Sirt1 modulates HSF1 activity, thereby influencing HSP expression, including *HSP70* and *HSP90* [57]. Here, *Sirt1*, *HSF1*, and *HSP* expression was significantly lower in the selected population than in the normal population under high temperature stress. These results suggest downregulation of the Sirt1/HSF1/HSP axis in the selected population under high temperature stress. This may reflect reduced reliance on the inducible heat shock response. Instead, enhanced thermotolerance may involve alternative protective mechanisms. Alternatively, lower *Sirt1* and *HSF1* expressions might result from reduced cellular stress burden rather than an active regulatory strategy.

Temperature stress significantly alters alternative splicing patterns in aquatic animals, and the SRSF family plays a critical regulatory role in this process [58]. Here, SRSF family genes showed low expression under control conditions but were induced by temperature stress, with distinct expression patterns between the two populations under high temperature. To date, studies on thermal adaptation from an alternative splicing perspective have largely focused on splicing pattern analyses; how temperature regulates alternative splicing via the SRSF family remains largely unexplored. In addition, qPCR validation results were consistent with RNA-seq expression patterns, confirming the reliability of the transcriptomic data.

Notably, the present conclusions regarding autophagy and E3-mediated regulation are derived exclusively from transcriptomic data. Functional validation at the protein level is required to confirm these transcriptional observations. Key approaches include measuring the LC3-II/I ratio to assess autophagic flux or assessing global ubiquitination levels. Thus, these findings should be interpreted as transcriptional evidence of potential mechanisms, pending experimental validation. Furthermore, SNPs located in regulatory elements such as promoters and enhancers can also significantly affect gene expression levels, thereby accounting for a portion of the observed expression differences. Consequently, our transcriptomic data cannot distinguish whether the expression differences arise from alterations in transcriptional regulation or from population genetic variation. We acknowledge that our libraries were constructed from pooled RNA samples, which averages out individual-level variation. Future studies using individual crayfish, especially extremely thermotolerant and sensitive individuals, are needed to resolve inter-individual heterogeneity. In addition, we acknowledge that one sample from the normal population (NP_35) was excluded from the transcriptomic analysis due to its outlier position in the PCA plot. Although this sample passed basic quality control, we could not rule out the possibility that it originated from an individual with mixed genetic background due to unintended mixing during aquaculture maintenance. Given the absence of genotypic markers (e.g., mitochondrial DNA or population-specific SNPs) to verify its origin, we decided to exclude it to avoid confounding the transcriptomic comparisons. Together, these findings suggest that heat tolerance in *P. clarkii* may be partially attributable to temperature-induced autophagic responses and transcription factor regulation rather than *HSP* accumulation. Notably, our experimental design did not include a recovery group (e.g., returning to 23 °C after temperature stress). Thus, transcriptional changes observed under heat or cold stress may reflect a mixture of active stress responses and accumulated damage rather than purely adaptive mechanisms. Future studies incorporating a recovery phase would help distinguish stress-induced injury from genuine repair processes, thereby providing a more comprehensive understanding of thermal adaptation in *P. clarkii*.

## 5. Conclusions

Here, we compared the abdominal ganglion transcriptomes of two *P. clarkii* populations with contrasting thermal tolerances. Low-temperature stress predominantly activated energy metabolism pathways (AMPK, PPAR, FoxO), whereas heat stress primarily impaired membrane-associated functions and disrupted redox homeostasis. In the selected population, heat stress significantly suppressed immune-related pathways (JAK-STAT, TGF-β, NLR) and reduced HSP, HSF1, and Sirt1 expression while upregulating autophagy-related genes (ATG5, LC3C). These findings suggest that temperature-responsive-related genes are involved in enhanced thermotolerance. This study provides a transcriptomic resource for understanding thermal adaptation in crustaceans and identifies candidate pathways and genes for functional validation and selective breeding of thermotolerant *P. clarkii* strains.

## Figures and Tables

**Table 1:** Summary of experimental conditions and sample groups.

Population	Temperature (°C)	Sample ID
Normal	23	NP_1, NP_2, NP_3
	8	NP_8_1, NP_8_2, NP_8_3
	35	NP_35_1, NP_35_2, NP_35_3
Selected	23	SP_1, SP_2, SP_3
	8	SP_8_1, SP_8_2, SP_8_3
	35	SP_35_1, SP_35_2, SP_35_3

**Table 2:** Summary of RNA-seq data quality for *P. clarkii* abdominal ganglion.

Sample	Raw Reads	Clean Reads	Error Rate (%)	Q30 (%)	GC Content (%)	Uniquely Mapped
SP_1	67,887,154	67,391,368	0.0118	96.36	41.58	54,018,459 (80.16%)
SP_2	75,586,436	75,044,908	0.0118	96.34	41.41	61,092,927 (81.41%)
SP_3	65,380,586	64,910,894	0.0117	96.40	42.29	48,491,649 (74.7%)
SP_8_1	61,200,388	60,709,410	0.0118	96.27	41.52	48,223,700 (79.43%)
SP_8_2	63,412,184	62,950,030	0.0118	96.22	41.61	51,561,995 (81.91%)
SP_8_3	66,740,376	66,199,840	0.0118	96.24	41.38	54,186,990 (81.85%)
NP_8_1	70,807,254	70,200,804	0.0119	96.12	42.37	56,557,360 (80.57%)
NP_8_2	77,724,526	77,101,734	0.0120	96.00	42.44	62,000,586 (80.41%)
NP_8_3	69,963,732	69,413,214	0.0119	96.10	42.36	54,717,976 (78.83%)
NP_1	63,160,956	62,684,830	0.0118	96.34	41.66	50,510,419 (80.58%)
NP_2	73,308,782	72,772,894	0.0119	96.14	41.85	59,098,059 (81.21%)
NP_3	72,510,570	71,935,158	0.0118	96.24	41.9	57,638,924 (80.13%)
NP_35_1	72,109,726	71,557,832	0.0119	96.2	42.09	56,848,009 (79.44%)
NP_35_2	64,810,086	64,308,832	0.0118	96.22	42.95	49,983,950 (77.72%)
NP_35_3	61,363,178	60,865,380	0.0118	96.26	41.69	47,549,725 (78.12%)
SP_35_1	64,742,380	64,222,854	0.0119	96.18	40.45	52,291,976 (81.42%)
SP_35_2	70,324,174	69,749,772	0.0119	96.15	40.9	56,906,352 (81.59%)
SP_35_3	68,121,464	67,593,618	0.0119	96.06	40.62	55,382,103 (81.93%)

Notes: Raw reads = Total number of raw sequencing reads generated per sample. Clean reads = Number of reads remaining after quality control. Error rate (%) = Expected sequencing error rate calculated from Phred scores; lower values indicate higher base-calling accuracy. Q30 (%) = Percentage of bases with a Phred quality score ≥ 30, corresponding to a base-calling accuracy of ≥99.9%. Q30 ≥ 90% is generally considered acceptable for downstream analysis. GC content (%) = Percentage of guanine and cytosine bases in the clean reads; reflects nucleotide composition and can indicate potential bias. Uniquely mapped = Number (and percentage) of clean reads that aligned uniquely to the reference genome (GCF_040958095.1). Higher uniquely mapped rates indicate better specificity and lower contamination or multi-mapping ambiguity.

## Data Availability

The raw RNA-seq data generated in this study have been deposited in the NCBI Sequence Read Archive (SRA) under accession number PRJNA1442656. All data can be freely downloaded and used for scientific research and analysis, subject to the database policies and relevant licensing agreements.

## Supplementary Materials

The following supporting information can be downloaded at: https://www.mdpi.com/article/10.3390/ani16131988/s1, Table S1: Primers used for qPCR.

## Abbreviations

The following abbreviations are used in this manuscript:

AMPK	AMP-Activated Protein Kinase
ATG	Autophagy-Related Gene
DEG	Differentially Expressed Gene
FoxO	Forkhead Box O
FOXO3	Forkhead Box O3
GO	Gene Ontology
HSF1	Heat Shock Factor 1
HSP	Heat Shock Protein
JAK-STAT	Janus Kinase-Signal Transducer and Activator of Transcription
KEGG	Kyoto Encyclopedia of Genes and Genomes
Klf	Krüppel-Like Factor
LC3C	Microtubule-Associated Protein 1 Light Chain 3 Gamma
LZTS2	Leucine Zipper Tumor Suppressor 2
MSL2	Male-Specific Lethal 2
NOD-like	Nucleotide-Binding Oligomerization Domain-Like
Pkd2	Polycystic Kidney Disease 2
PPAR	Peroxisome Proliferator-Activated Receptor
RNF220	Ring Finger Protein 220
SCE	Sister Chromatid Exchange
Sirt	Sirtuin
SRSF	Serine/Arginine-Rich Splicing Factor
TGF-β	Transforming Growth Factor Beta
TRP	Transient Receptor Potential
TRPA	Transient Receptor Potential Ankyrin
TRPC	Transient Receptor Potential Canonical
UBR4l	Ubiquitin Protein Ligase E3 Component N-Recognin 4-like
ULK1	Unc-51 Like Autophagy Activating Kinase 1

## References

[1] Wang Q., Jia L., Yang B., Liu Y., Bai Z. (2026). Thermal adaptation strategies in crustaceans: Potential threats to aquaculture in a warming climate. Aquac. Fish..

[2] Qian Y., Yu Q., Zhang J., Han Y., Xie X., Zhu D. (2025). Molecular characterization of TRPA1 and its function in thermotaxis and molting in *Portunus trituberculatus*. Aquac. Rep..

[3] Li R., Qi J., Hu L., Huang J., Yang J., Lin R., Sun J. (2023). Molecular characterization of TRPA1 and its function in temperature preference in *Eriocheir sinensis*. Comp. Biochem. Physiol. Part A Mol. Integr. Physiol..

[4] Xia B., Song J., Xin Q., Wang C., Yu W., Liu J., Yu G., Wang F., Xu D. (2026). Mechanisms of heat and hypoxia defense in the sea cucumber *Apostichopus japonicus*: Insights from ubiquitination regulation. Comp. Biochem. Physiol. Part D Genom. Proteom..

[5] Wang J.-Y., Yu Y., Zhou F.-L., Shi J.-Z., Huang J.-H., Li Y.-D., Yang L.-S., Liang J.-Q., Yang Q.-B., Jiang S. (2025). Effects of Temperature and Salinity on Tissue Structure, Antioxidant Capacity, and Heat-Shock Protein Gene Expression in *Penaeus monodon*. Russ. J. Mar. Biol..

[6] Molina A., Dettleff P., Valenzuela-Muñoz V., Gallardo-Escarate C., Valdés J.A. (2023). High-temperature stress induces autophagy in rainbow trout skeletal muscle. Fishes.

[7] Li Q.-Q., Zhang J., Wang H.-Y., Niu S.-F., Wu R.-X., Tang B.-G., Wang Q.-H., Liang Z.-B., Liang Y.-S. (2023). Transcriptomic response of the liver tissue in *Trachinotus ovatus* to acute heat stress. Animals.

[8] Jeyachandran S., Chellapandian H., Park K., Kwak I.-S. (2023). A review on the involvement of heat shock proteins (extrinsic chaperones) in response to stress conditions in aquatic organisms. Antioxidants.

[9] Luling R., John H., Gudermann T., Thiermann H., Muckter H., Popp T., Steinritz D. (2018). Transient Receptor Potential Channel A1 (TRPA1) Regulates Sulfur Mustard-Induced Expression of Heat Shock 70 kDa Protein 6 (HSPA6) In Vitro. Cells.

[10] Iftinca M., Flynn R., Basso L., Melo H., Aboushousha R., Taylor L., Altier C. (2016). The stress protein heat shock cognate 70 (Hsc70) inhibits the Transient Receptor Potential Vanilloid type 1 (TRPV1) channel. Mol. Pain.

[11] Bromberg Z., Goloubinoff P., Saidi Y., Weiss Y.G. (2013). The membrane-associated transient receptor potential vanilloid channel is the central heat shock receptor controlling the cellular heat shock response in epithelial cells. PLoS ONE.

[12] Fan L., Wang A., Wu Y. (2013). Comparative proteomic identification of the hemocyte response to cold stress in white shrimp, *Litopenaeus vannamei*. J. Proteom..

[13] Hossain M.S., Zaman B., Khan M.A., Shihab M.Y.K., Nielsen R. (2025). Impact of climate change and adaptation strategy in aquaculture: A systematic review. Aquac. Int..

[14] National Aquatic Technology Extension Station, China Society of Fisheries (2025). China Crayfish Industry Development Report (2025).

[15] Yadav N.K., Patel A.B., Singh S.K., Mehta N.K., Anand V., Lal J., Dekari D., Devi N.C. (2024). Climate change effects on aquaculture production and its sustainable management through climate-resilient adaptation strategies: A review. Environ. Sci. Pollut. Res..

[16] Ding Y., Sha W., Sun Y., Cheng Y. (2025). Effects of acute low-temperature stress on respiratory metabolism, antioxidants, and metabolomics of red swamp crayfish, *Procambarus clarkii*. Comp. Biochem. Physiol. Part B Biochem. Mol. Biol..

[17] Chen X., Huang L., Chen J., Wu B., Wu C., Mei Y., He J. (2025). Transcriptome analysis of muscles reveals the effect of low temperature on the growth of *Procambarus clarkii*. Aquac. Rep..

[18] Zhu X., Ren X., Xiong L., Liu T., Bai X. (2024). Genetic dissection of crayfish (*Procambarus clarkii*) high temperature tolerance and assessment of the potential application in breeding of the HSP genes. Comp. Biochem. Physiol. Part D Genom. Proteom..

[19] Xiao R., Xu X.S. (2021). Temperature sensation: From molecular thermosensors to neural circuits and coding principles. Annu. Rev. Physiol..

[20] Weijie W., Qizhou X., Tong W., Liang J., Xi C., Dianzhong L., Zhiyi B. (2024). Comparison of high-temperature tolerance in three diallel hybrid populations of red swamp crayfish (*Procambarus clarkii*). J. Dalian Ocean Univ..

[21] Frederich M., Pörtner H.O. (2000). Oxygen limitation of thermal tolerance defined by cardiac and ventilatory performance in spider crab, *Maja squinado*. Am. J. Physiol.-Regul. Integr. Comp. Physiol..

[22] Marshall D.J., Dong Y.-w., McQuaid C.D., Williams G.A. (2011). Thermal adaptation in the intertidal snail *Echinolittorina malaccana* contradicts current theory by revealing the crucial roles of resting metabolism. J. Exp. Biol..

[23] Chen S., Zhou Y., Chen Y., Gu J. (2018). fastp: An ultra-fast all-in-one FASTQ preprocessor. Bioinformatics.

[24] Kim D., Paggi J.M., Park C., Bennett C., Salzberg S.L. (2019). Graph-based genome alignment and genotyping with HISAT2 and HISAT-genotype. Nat. Biotechnol..

[25] Li B., Dewey C.N. (2011). RSEM: Accurate transcript quantification from RNA-Seq data with or without a reference genome. BMC Bioinform..

[26] Love M.I., Huber W., Anders S. (2014). Moderated estimation of fold change and dispersion for RNA-seq data with DESeq2. Genome Biol..

[27] Klopfenstein D.V., Zhang L., Pedersen B.S., Ramírez F., Warwick Vesztrocy A., Naldi A., Mungall C.J., Yunes J.M., Botvinnik O., Weigel M. (2018). GOATOOLS: A Python library for Gene Ontology analyses. Sci. Rep..

[28] Bu D., Luo H., Huo P., Wang Z., Zhang S., He Z., Wu Y., Zhao L., Liu J., Guo J. (2021). KOBAS-i: Intelligent prioritization and exploratory visualization of biological functions for gene enrichment analysis. Nucleic Acids Res..

[29] Haberberger R.V., Barry C., Dominguez N., Matusica D. (2019). Human dorsal root ganglia. Front. Cell. Neurosci..

[30] Jia Z., Ling J., Gu J.G. (2012). Temperature dependence of rapidly adapting mechanically activated currents in rat dorsal root ganglion neurons. Neurosci. Lett..

[31] Neelands T., Jarvis M., Faltynek C., Surowy C. (2008). Elevated temperatures alter TRPV1 agonist-evoked excitability of dorsal root ganglion neurons. Inflamm. Res..

[32] Yang Y., Xu W., Jiang Q., Ye Y., Tian J., Huang Y., Du X., Li Y., Zhao Y., Liu Z. (2022). Effects of low temperature on antioxidant and heat shock protein expression profiles and transcriptomic responses in crayfish (*Cherax destructor*). Antioxidants.

[33] Luo L., Huang J.H., Liu D.L., Jiang S.G., Zhou F.L., Jiang S., Yang Q.B., Li Y.D., Li T., Tan L.Q. (2021). Transcriptome reveals the important role of metabolic imbalances, immune disorders and apoptosis in the treatment of *Procambarus clarkii* at super high temperature. Comp. Biochem. Physiol. Part D Genom. Proteom..

[34] Andreassen A.H., Clements J.C., Morgan R., Spatafora D., Metz M., Åsheim E.R., Pélabon C., Jutfelt F. (2025). Evolution of warming tolerance alters physiology and life history traits in zebrafish. Nat. Clim. Change.

[35] Wu D., Liu Z., Yu P., Huang Y., Cai M., Zhang M., Zhao Y. (2020). Cold stress regulates lipid metabolism via AMPK signalling in *Cherax quadricarinatus*. J. Therm. Biol..

[36] Frederich M., O’Rourke M.R., Furey N.B., Jost J.A. (2009). AMP-activated protein kinase (AMPK) in the rock crab, *Cancer irroratus*: An early indicator of temperature stress. J. Exp. Biol..

[37] Zhao T., Ma A., Huang Z., Liu Z., Sun Z., Wang X., Xu R. (2022). Structural and functional characterization of turbot pparγ: Activation during high temperature and regulation of lipid metabolism. J. Therm. Biol..

[38] Ament Z., West J.A., Stanley E., Ashmore T., Roberts L.D., Wright J., Nicholls A.W., Griffin J.L. (2016). PPAR-pan activation induces hepatic oxidative stress and lipidomic remodelling. Free Radic. Biol. Med..

[39] Milan G., Romanello V., Pescatore F., Armani A., Paik J.-H., Frasson L., Seydel A., Zhao J., Abraham R., Goldberg A.L. (2015). Regulation of autophagy and the ubiquitin–proteasome system by the FoxO transcriptional network during muscle atrophy. Nat. Commun..

[40] Kim D.H., Perdomo G., Zhang T., Slusher S., Lee S., Phillips B.E., Fan Y., Giannoukakis N., Gramignoli R., Strom S. (2011). FoxO6 integrates insulin signaling with gluconeogenesis in the liver. Diabetes.

[41] Jing Z., Chen Q., Yan C., Zhang C., Xu Z., Huang X., Wu J., Li Y., Yang S. (2023). Effects of chronic heat stress on kidney damage, apoptosis, inflammation, and heat shock proteins of Siberian sturgeon (*Acipenser baerii*). Animals.

[42] Keestra-Gounder A.M., Byndloss M.X., Seyffert N., Young B.M., Chávez-Arroyo A., Tsai A.Y., Cevallos S.A., Winter M.G., Pham O.H., Tiffany C.R. (2016). NOD1 and NOD2 signalling links ER stress with inflammation. Nature.

[43] Fowler M.A., Montell C. (2013). Drosophila TRP channels and animal behavior. Life Sci..

[44] Liénard M.A., Baez-Nieto D., Tsai C.-C., Valencia-Montoya W.A., Werin B., Johanson U., Lassance J.-M., Pan J.Q., Yu N., Pierce N.E. (2024). *TRPA5* encodes a thermosensitive ankyrin ion channel receptor in a triatomine insect. iScience.

[45] Turner H.N., Armengol K., Patel A.A., Himmel N.J., Sullivan L., Iyer S.C., Bhattacharya S., Iyer E.P.R., Landry C., Galko M.J. (2016). The TRP Channels Pkd2, NompC, and Trpm Act in Cold-Sensing Neurons to Mediate Unique Aversive Behaviors to Noxious Cold in Drosophila. Curr. Biol..

[46] Neely G.G., Keene A.C., Duchek P., Chang E.C., Wang Q.-P., Aksoy Y.A., Rosenzweig M., Costigan M., Woolf C.J., Garrity P.A. (2011). TrpA1 regulates thermal nociception in Drosophila. PLoS ONE.

[47] Sokabe T., Tsujiuchi S., Kadowaki T., Tominaga M. (2008). Drosophila Painless Is a Ca^2+^-Requiring Channel Activated by Noxious Heat. J. Neurosci..

[48] Ma X., Gu W., Yang C., He Z., Fan H., Wang L., Song L. (2024). Inhibition of TRPA1-like alleviated unfolded protein response and apoptosis by regulating cytoplasmic Ca^2+^ in Yesso scallop *Patinopecten yessoensis* under high temperature stress. Front. Mar. Sci..

[49] Pirkkala L., Nykänen P., Sistonen L. (2001). Roles of the heat shock transcription factors in regulation of the heat shock response and beyond. FASEB J..

[50] Liu Y., Xiao S., Sun H., Pei L., Liu Y., Peng L., Gao X., Liu Y., Wang J. (2020). AtPPRT1, an E3 ubiquitin ligase, enhances the thermotolerance in Arabidopsis. Plants.

[51] Peng L., Wan X., Huang K., Pei L., Xiong J., Li X., Wang J. (2019). AtPUB48 E3 ligase plays a crucial role in the thermotolerance of Arabidopsis. Biochem. Biophys. Res. Commun..

[52] Iriondo M.N., Etxaniz A., Varela Y.R., Ballesteros U., Lázaro M., Valle M., Fracchiolla D., Martens S., Montes L.R., Goñi F.M. (2023). Effect of ATG12–ATG5-ATG16L1 autophagy E3-like complex on the ability of LC3/GABARAP proteins to induce vesicle tethering and fusion. Cell. Mol. Life Sci..

[53] Lystad A.H., Carlsson S.R., Simonsen A. (2019). Toward the function of mammalian ATG12–ATG5-ATG16L1 complex in autophagy and related processes. Autophagy.

[54] Nie P., Wang Y., Wang Y., Xi B., Wei B., Shang S., Dewer Y. (2026). TuATG1-mediated autophagy confers thermotolerance in *Tetranychus urticae* and provides an RNAi target for pest management. Pest Manag. Sci..

[55] Zhou Jie Z.J., Wang Jian W.J., Yu JingQuan Y.J., Chen ZhiXiang C.Z. (2014). Role and regulation of autophagy in heat stress responses of tomato plants. Front. Plant Sci..

[56] O’Brien K., Cominassi L., Robine S., Marbacher P., Ressel K. (2025). Sirtuins may mediate temperature-induced metabolic remodeling in threespine stickleback. J. Comp. Physiol. B.

[57] Raynes R., Pombier K.M., Nguyen K., Brunquell J., Mendez J.E., Westerheide S.D. (2013). The SIRT1 modulators AROS and DBC1 regulate HSF1 activity and the heat shock response. PLoS ONE.

[58] Chan S.K.N., Suresh S., Munday P., Ravasi T., Bernal M.A., Schunter C. (2022). The alternative splicing landscape of a coral reef fish during a marine heatwave. Ecol. Evol..

